# Cannabidiol effectively prevents oxidative stress and stabilizes hypoxia-inducible factor-1 alpha (HIF-1α) in an animal model of global hypoxia

**DOI:** 10.1038/s41598-024-66599-5

**Published:** 2024-07-10

**Authors:** Hanna Kletkiewicz, Michał S. Wojciechowski, Justyna Rogalska

**Affiliations:** 1 Department of Animal Physiology and Neurobiology, Faculty of Biological and Veterinary Sciences, Nicolaus Copernicus University, Lwowska 1, 87–100 Toruń, Poland; 2 Centre For Modern Interdisciplinary Technologies, Nicolaus Copernicus University, Wileńska 4, 87–100 Toruń, Poland; 3 Department of Vertebrate Zoology, Faculty of Biological and Veterinary Sciences, Nicolaus Copernicus University, Lwowska 1, 87–100 Toruń, Poland

**Keywords:** Perinatal hypoxia, Cannabidiol, Oxidative stress, Lipid peroxidation, Antioxidants, Hypoxia-inducible factor-1 alpha, Neuroscience, Physiology, Diseases

## Abstract

Cannabidiol (CBD) is a non-psychotomimetic phytocannabinoid derived from *Cannabis sativa*. It has therapeutic effects in different paradigms of brain injury, acting as a neuroprotectant. As oxidative stress is a primary risk factor for brain damage after neonatal hypoxia, we tested the effect of CBD on oxidative status and non-protein-bound iron accumulation in the immature brain after hypoxia. Moreover, we tested whether cannabidiol affects the accumulation of hypoxia-inducible factor-1 alpha (HIF-1α) which plays a key role in the regulation of cellular adaptation to hypoxia and oxidative stress. We used 7-day-old mice randomly assigned to hypoxic or control groups. Immediately after hypoxia or control exposure, pups were randomly assigned to a vehicle or CBD treatment. 24 h later, they were decapitated and the brains were immediately removed and stored for further biochemical analyses. We found that CBD reduced lipid peroxidation and prevented antioxidant depletion. For the first time, we also demonstrated that CBD upregulated HIF-1α protein level. This study indicates that CBD may effective agent in attenuating the detrimental consequences of perinatal asphyxia.

## Introduction

Perinatal asphyxia resulting in hypoxic brain injury remains an important problem in modern obstetrics. The incidence of asphyxia is approximately 1.5 cases per 1000 live births in the developed countries and from 10 to 20 per 1000 live births in the low and middle-income countries^1,2^. During the last two decades, many studies have searched for the potential therapeutic strategies for treatment or preventing detrimental consequences of hypoxia^3^. However, except for the therapeutic hypothermia (TH), so far, no other therapies exist. While TH is clearly beneficial in preventing death, it does not provide complete neuroprotection^4^. Further attempts to refine TH suggest that current cooling protocols are near-optimal^5^ thus additional neuroprotective therapies are still needed.

One of the most important factors involved in hypoxic brain injury is the accumulation of reactive oxygen species (ROS) which shift the balance between oxidants and antioxidants leading to oxidative stress^6,7^. Oxidative stress is particularly harmful to the neonatal brain because of its high oxygen consumption, high contents of unsaturated fatty acids and water, low myelination, low concentrations of antioxidants, and low availability of redox-active iron^8^. In response to hypoxia-induced oxidative stress, glial cells, particularly astrocytes and microglia, have the potential to release large amounts of ROS^9^. Additionally, astrocytes can contribute to ROS production indirectly by promoting inflammation and by regulating the activity of other cell types, including microglia. When activated, microglia release pro-inflammatory molecules and generate ROS as part of their defense mechanism. This ROS production serves to eliminate pathogens and damaged cells. However, excessive ROS production by activated microglia can contribute to neuroinflammation and neuronal damage^9^. Oxidative stress initiated by hypoxia and subsequent reoxygenation lead to lipid and protein oxidation and DNA degeneration^10,11^. Several studies showed a direct association between the degree of hypoxia and the severity of oxidative stress in the neonatal period^12,13^. Thus, effective hypoxia treatment in neonates should be directed also toward the improvement of its antioxidant capacity.

One of the promising antioxidant agents is cannabidiol (CBD), the main non-psychoactive component of *Cannabis sativa*^14^. Due to its lipophilic nature, CBD accumulates in cell membranes^15^, which makes it a particularly effective antioxidant for the membrane components. Up to now, the antioxidant potential of CBD was demonstrated in cancer, cardiovascular, neurodegenerative, and metabolic diseases, which are usually accompanied by oxidative stress^16,17^. Recent studies showed that CBD can the affect redox balance by modifying the level and activity of antioxidants and oxidants independently from the classical cannabinoid receptors^15^. CBD may increase the amount and activity of antioxidant enzymes^18–20^ and interrupt the free radical chain reactions^21^. Moreover, CBD prevents the oxidation of glutathione (GSH) and other non-enzymatic antioxidants^19^, and chelates non-protein-bound metal ions involved in the Fenton reaction^22^. However, whether chelating function of CBD plays a neuroprotective role in hypoxic immature brains is unknown.

**Figure 1:**
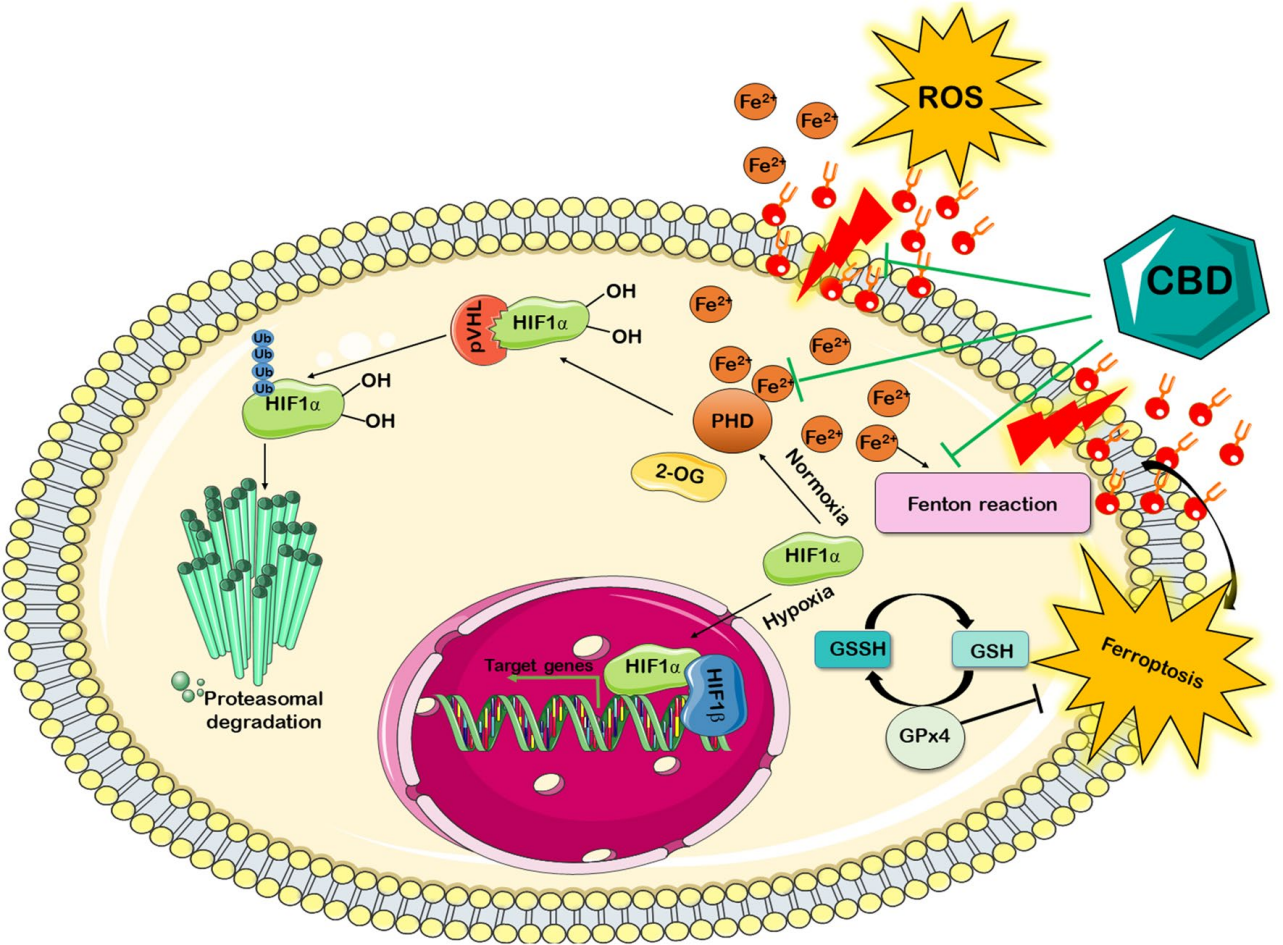
Schematic illustration of the hypoxia-inducible factor-1 alpha (HIF-1α), iron ions, and cannabidiol (CBD) interactions. Under normoxia, HIF-1α is hydroxylated by prolyl-4-hydroxylases (PHD). The hydroxylation is catalyzed using Fe^2+^ as co-factors and 2-oxoglutarate (2-OG). Next, HIF-1α is polyubiquitinated by the von Hippel–Lindau protein (pVHL) and degraded in the proteasome. In response to hypoxia or PHD inhibitors, HIF-1α is stabilized and translocated into the nucleus, where it binds to its dimerization partner HIF-1β and enhances the transcription of HIF target genes. During hypoxia, iron liberated from iron-storage proteins pool generates reactive oxygen species (ROS) through the Fenton reaction, leading to lipid peroxidation. The excessive lipid peroxidation triggers ferroptosis. Ferroptosis can be inhibited by glutathione peroxidase 4 (GPx4). This reaction occurs through the use of glutathione (GSH) as a substrate. The antioxidant properties of the CBD has been highlighted by black lines.

During hypoxia, iron can be liberated from the iron-storage proteins pool to produce free radicals, and free radicals, in turn, can induce the release of more iron (Fig. 1). Moreover, non-protein-bound iron may also increase the activity of iron-containing enzymes such as prolyl hydroxylase (PHD)—Dependent proteasomal system^23^, which plays a key role in the degradation of hypoxia-inducible factor-1 alpha (HIF-1α)^24^. HIF-1α is a transcriptional activator and a key regulator of the cellular adaptive responses to hypoxia and oxidative stress^25,26^. Under normoxic conditions, HIF-1α is rapidly degraded by PHD, but it accumulates when the PHD activity is repressed by low oxygen tension or iron chelators. Upon its accumulation and phosphorylation, HIF-1α dimerizes with HIF-β (constitutively expressed nuclear proteins), enters the nucleus, and promotes the transcription of hypoxia response elements (HRE)-containing target genes involved in energy metabolism, erythropoiesis, vascular remodelling, and other processes (Fig. 1). HIF-1α also regulates many genes involved in the iron homeostasis as well as promotes fatty acid uptake and lipid storage by transcriptional upregulation of fatty acid-binding proteins^26^. Thus, there are strong theoretical underpinnings for studies on CBD as a powerful tool to stabilize HIF-1α protein in the brain after neonatal hypoxia.

In the present study, we asked whether CBD may prevent hypoxia-induced oxidative stress, antioxidant depletion, and non-protein-bound iron accumulation. Moreover, we asked whether CBD affects HIF-1α accumulation. To answer the first question, we measured malondialdehyde (MDA) level – the product of the brain aldehydic lipid peroxidation and 8-epi Prostaglandin F2α (8-epi PGF2α) concentration – a marker of oxidative stress and antioxidant deficiency. To answer the second question we measured glutathione (GSH) level and total antioxidant capacity (TAC). Finally, to answer the last two questions, we examined the iron content and the HIF-1α protein level in the hippocampal area of the mice brain. We chose the hippocampus because it is particularly vulnerable to perinatal hypoxia^27,28^.

## Results

### Effect of hypoxia and CBD treatment on oxidative stress and antioxidant capacity

**Figure 2:**
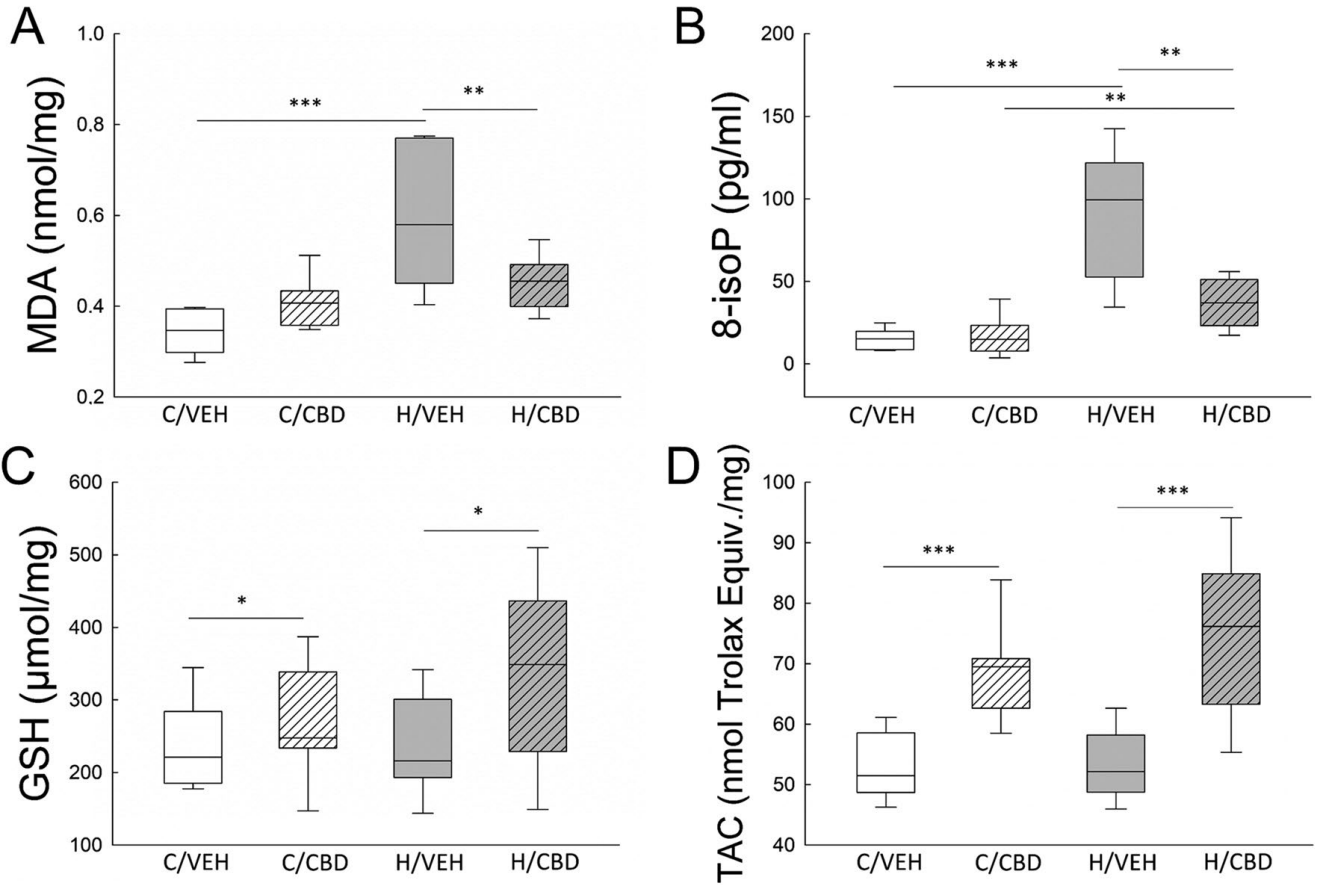
Effect of hypoxia and CBD treatment on oxidative stress and antioxidant capacity in neonatal mice brain. The concentration of MDA (**A**), 8-epi PGF2α (**B**), GSH (**C**), and TAC (**D**) in the control vehicle group (C/VEH), hypoxic vehicle group (H/VEH), control CBD group (C/CBD) and hypoxic CBD group (H/CBD) (*p < 0.5, **p < 0.01, ***p ≤ 0.001). The bottom and top edges of the box indicate the 25th and 75th percentiles, the solid line indicates the median and the whiskers extend to the most extreme data points not considered outliers.

Oxygen concentration and drug treatment significantly affected MDA level (F (1, 25) = 9.633; p = 0.005). Saline-injected mice had higher MDA level after exposure to hypoxia compared to normoxic mice (H/VEH 0.586 ± 0.032 nmol mg^–1^ vs. C/VEH 0.349 ± 0.032 nmol mg^–1^; p ≤ 0.001). CBD treatment attenuated the hypoxia-induced increase of lipid peroxidation (H/VEH 0.586 ± 0.032 nmol mg^–1^ vs H/CBD 0.454 ± 0.030 nmol mg^–1^, p < 0.01). There was no significant difference in lipid peroxidation between the control groups (C/VEH vs C/CBD). In hypoxic animals treated with CBD (H/CBD, 0.454 ± 0.030 nmol mg^–1^) MDA level was slightly higher than that found in their control counterparts (C/VEH, 0.408 ± 0.032 nmol mg^–1^), but this difference did not reach the level of significance (Fig. 2A).

The 8-epi PGF2α concentration was also affected by oxygen conditions and drug treatment (F (1, 25) = 4.357; p = 0.047). Hypoxia itself induced a sixfold increase in 8-epi PGF2α level (C/VEH 15.264 ± 8.039 pg mg^–1^ vs. H/VEH 92.893 ± 8.039 pg mg^–1^, p ≤ 0.001). CBD treatment after hypoxia exposure decreased 8-epi PGF2α more than twice compared to the vehicle-treated hypoxic mice (H/VEH 92.893 ± 8.039 pg mg^–1^ vs. H/CBD 36.913 ± 7.520 pg mg^–1^; p ≤ 0.001). However, the 8-epi PGF2α level in hypoxic mice treated with CBD was still higher than that in the control group (H/CBD 36.913 ± 7.520 pg mg^–1^ vs C/CBD 16.769 ± 8.039 pg mg^–1^, p < 0.01). There was no difference in 8-epi PGF2α between both control groups (Fig. 2B).

In general, there was no difference between control and hypoxic animals in GSH and TAC content (F (1, 25) = 0.877; p = 0.358 and F (1, 25) = 1.003; p = 0.326, respectively). However, GSH and TAC levels were affected by drug treatment (F (1, 25) = 33.079; p ≤ 0.001 and F (1, 25) = 4.562; p < 0.05, respectively) and were significantly higher in mice receiving CBD than in vehicle animals (Fig. 2C and D).

**Figure 3:**
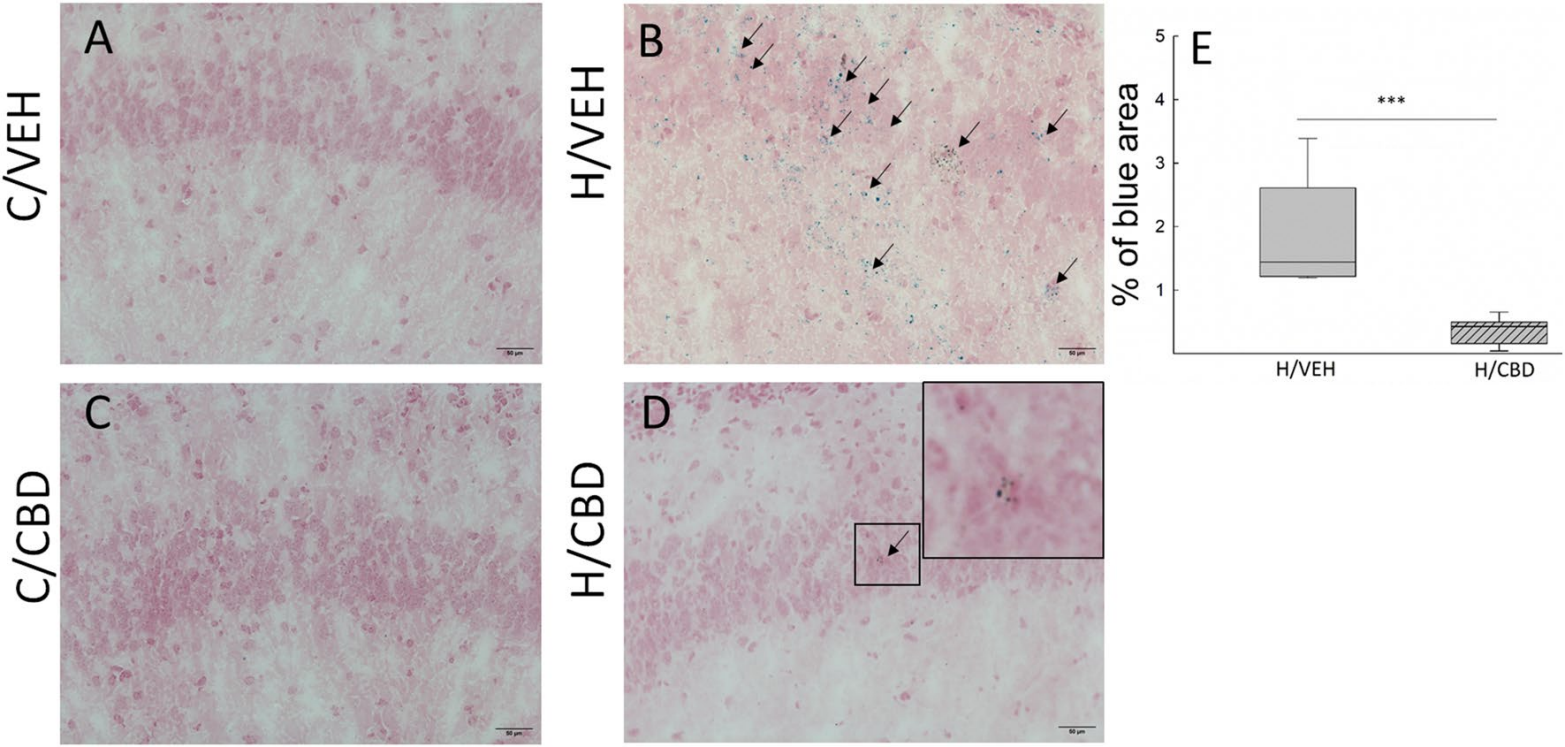
Effects of hypoxia and CBD treatment on free iron accumulation. Representative images of iron staining in the hippocampus area of the (**A**) control vehicle group (C/VEH); (**B**) hypoxic vehicle group (H/VEH); (**C**) control CBD group (C/CBD) and (**D**) hypoxic CBD group (H/CBD). No iron-positive cells were observed in the brain sections of both control groups (panels (**A**) and (**C**)). Iron deposition was the most obvious in the hypoxic vehicle group (panel (**B**)). A small amount of iron deposits has been observed in the hypoxic CBD group (panel (**D**)). Scale bars: 50 μm; staining is denoted by the arrows. Panel (**E**)—A quantitative evaluation using Fiji software shows the percentage of blue spots in hypoxic groups (***p ≤ 0.001). Box indicates 25th and 75th percentiles, solid line stands for the median in each experimental group.

CBD treatment attenuated iron accumulation in hypoxic mice (Fig. 3D and E, p ≤ 0.001). Control animals showed no free iron deposits in the examined brain area (Fig. 3A and C).

### Effect of hypoxia and CBD treatment on HIF-1α concentration

**Figure 4:**
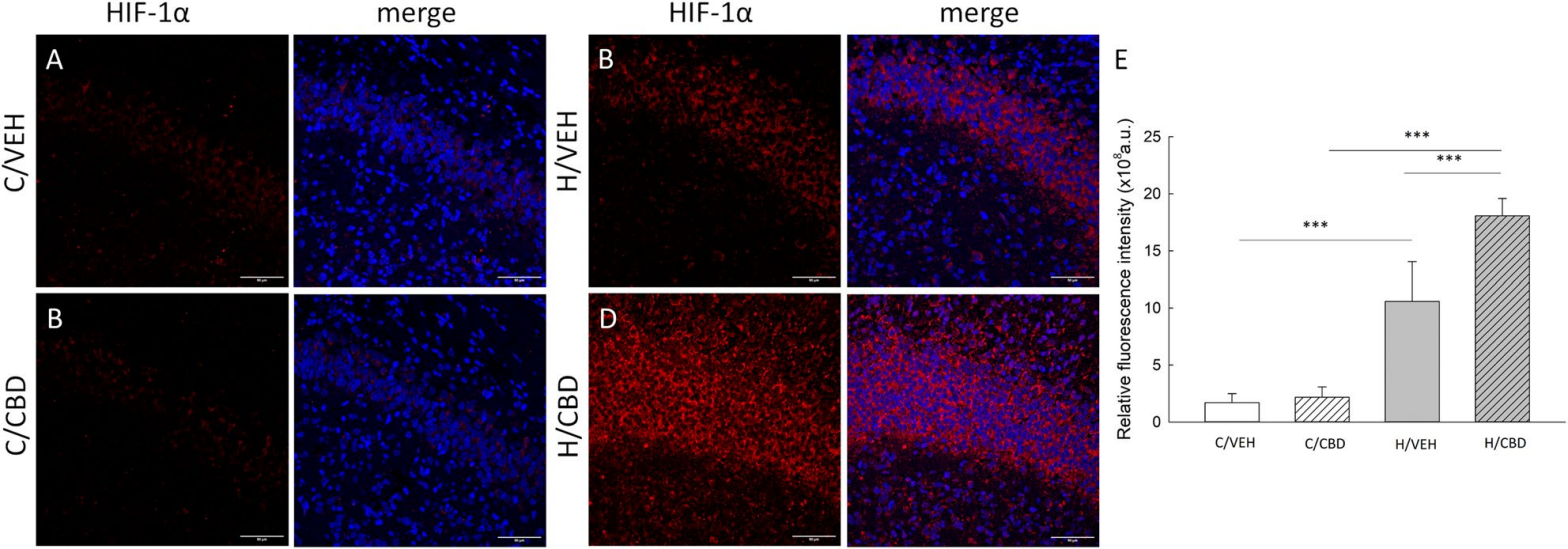
HIF-1α immunolocalization in brain sections of neonatal mice. Confocal images showing co-localization of HIF-1α (red) and Hoechst (merge) in the hippocampus area of the (**A**) control vehicle group (C/VEH); (**B**) hypoxic vehicle group (H/VEH); (**C**) control CBD group (C/CBD) and (**D**) hypoxic CBD group (H/CBD). In both control groups (panels (**A**) and (**C**)), the fluorescence was relatively weak. Scale bars: 50 µm. Panel (**E**)—A quantitative analysis of HIF-1α level in the neonatal mice hippocampus. Relative fluorescence intensity is expressed in arbitrary units (a.u). Values are presented as means ± SEM and ***p ≤ 0.001.

Hypoxia-induced an increase in HIF-1α level, both in vehicle- and CBD-treated mice compared to these detected in control animals (Fig. 4A–D) (H/VEH 1.06 × 10^9^ ± 8.80 × 10^7^ a.u. vs C/VEH 1.70 × 10^8^ ± 8.80 × 10^7^ a.u., p ≤ 0.001 and H/CBD 1.81 × 10^9^ ± 8.0410^7^ a.u. vs C/CBD 2.18 × 10^8^ ± 8.80 × 10^7^ a.u., p ≤ 0.001; respectively; Fig. 3E). However, the effect of CBD treatment on HIF-1α level depended on oxygen conditions (F (1, 17) = 16.509; p ≤ 0.001), namely the effect of CBD was recorded only in hypoxic animals (Fig. 4E).

## Discussion

Oxidative stress is a primary risk factor for brain damage after neonatal hypoxia. The main finding of our study is that cannabidiol (CBD) exerts an antioxidative effect in two ways. First, the protective action of CBD was achieved by a reduction of lipid peroxidation, an increase in antioxidants concentration, and suppression of non-protein-bound iron accumulation. Second, the effect of CBD was associated with the increase of hypoxia-inducible factor-1 alpha (HIF-1α) protein level, a master regulator of oxygen homeostasis and iron homeostasis^29^ (Fig. 1). To the best of our knowledge, this is the first report showing that the post-hypoxia treatment with CBD affects the HIF-1α level in the brain tissue.

Compared to other classes of lipids, polyunsaturated fatty acids (PUFAs), which are extremely abundant in the neonatal brain, are the most susceptible lipids to peroxidation^30^. The cascade of peroxidation reactions of PUFAs leads to the formation of lipid oxidation products, among which MDA and 8-epi PGF2α predominate^31^. Consistently with previous reports^32–36^, we found that MDA and 8-epi PGF2α concentrations were markedly higher in the hypoxic groups (Fig. 2). CBD administration in hypoxic mice significantly decreased concentration of both lipid peroxidation markers. Other studies have also shown that CBD treatment can reduce lipid peroxidation in C57BL/6 J mouse liver or in mouse neuronal cells (HT22) under reperfusion conditions^36^. According to the literature, this effect may result from CBD action interrupting the free radical chain reactions^37^ or chelating the transition metal ions involved in the Fenton reaction^22^. We observed that CBD attenuated the hypoxia-induced increase in free iron content (Fig. 3). Collectively, our results support the hypothesis that CBD effectively prevents hypoxia-induced lipid peroxidation.

We also found that CBD may also prevent hypoxia-induced antioxidant depletion. Glutathione (GSH), the most abundant intracellular antioxidant, is depleted under acute and chronic oxidative stress^38^. Previously, it was found that GSH level is reduced after hypoxia in the neonatal brain^39,40^ and in primary cultured oligodendrocytes exposed to hypoxia, or conditioned medium from hypoxic microglial cells^39^. Although in our study GSH level did not differ between hypoxic and normoxic animals, it was higher in mice treated with CBD (Fig. 2). This suggests that CBD modifies the redox balance by changing the level and activity of endogenous antioxidants. We also observed a significant increase in TAC concentration in animals treated with CBD (Fig. 2). It is consistent with the literature suggesting that CBD's antioxidant activity may involve activating the redox-sensitive nuclear erythroid 2-related factor (Nrf2)^41^ which plays a pivotal role in activating the endogenous antioxidant defense system^42^.

However, our study has several limitations. The lack of direct characterization of the glial compartment, the main sources of reactive oxygen species (ROS), limits our conclusions of CBD's effects on the central nervous system (CNS). Based on our results we cannot determine whether CBD directly affects CNS or if its effects are mediated through peripheral responses. We also cannot distinguish between potential cell-autonomous and non-cell-autonomous CBD effects. CBD can directly influence various cell types, including neurons and immune cells, by targeting distinct molecular pathways, thereby modulating their functions. CBD can also indirectly influence cellular activity by altering the release of neurotransmitters or cytokines from neighbouring cells, leading to non-cell autonomous effects. These points need further investigation.

The second arm of the neuroprotective action of CBD is related to the hypoxia-inducible factor-1 alpha (HIF-1α). HIF-1α level is regulated through different mechanisms, including its stabilization, phosphorylation, modifications of redox conditions, and interactions with co-activators^43^. Its half-life in normoxia is very short, typically less than 5 minutes^44^, but it accumulates in response to hypoxia within minutes to even 24 hours^45^. In the current study, CBD augmented an increase in HIF-1α levels recorded 24 h after exposure to hypoxia, likely by chelating transition metal ions^22^ that inhibit the enzyme responsible for HIF-1α degradation^46,47^. We suggest that the increase of HIF-1α level and decrease in lipid peroxidation in hypoxic mice treated with CBD, result from the reduction in free iron content in the hippocampus. In non-CBD treated mice, hypoxia-induced HIF-1α activation was less pronounced, while the lipid peroxidation and free iron level in the hippocampus were elevated. The localization of HIF-1α was mainly cytoplasmic in both hypoxic group, what is consistent with the observation in hypoxic differentiated U937 cells^48^. This significant cytoplasmic accumulation of HIF-1α appears to be a result of enhanced protein stabilization before its translocation to the nucleus. However, the HIF-1α translocation between cytoplasm and nucleus can vary over time. Future studies with multiple time-points and different methods are needed to fully understand the temporal aspects of HIF-1α localization and translocation. First, the cytoplasmic level of HIF-1α at single time-point does not allow for the conclusions on the dynamic changes in its distribution. Second, the HIF-1α detection may depend on the fixative used. Acetone, which we used in our study, is a commonly used fixative agent but, its effectiveness can vary depending on the specific antigen being studied and the overall experimental conditions.

Finally, although iron is involved in the development of oxidative cell death^49^ we did not find any neuron damage in the hippocampus (data not shown). This could be due to the timing of measurements, i.e. 24 h after hypoxia, while the neuronal cell death can occur days to weeks after hypoxic insult^50^. The lack of neuron damage in our study may also be due to the white matter's higher vulnerability compared to grey matter and the ongoing maturation of oligodendrocytes in 7-day-old mice^51^. Thus, the lack of morphological differences seems to result from the time of measurements.

## Concluding remarks

Our results show that CBD applied in a short time after hypoxia attenuates hypoxia-induced oxidative stress, likely due to its antioxidant activity. To the best of our knowledge, this is also the first report showing that the post-hypoxia treatment with CBD increases the concentration of HIF-1α, which is directly involved in the maintenance of oxygen and iron homeostasis. This indicates that CBD is promising agent for new therapies developed for the treatment of hypoxic injury However, further research is needed to fully understand the mechanisms of CBD's neuroprotective effects.

## Methodology

### Ethical approval

All animal experimentation was carried out in accordance with the European Communities Council Directive (86/609/EEC) and in accordance with Polish law on the care of laboratory animals. The experimental protocol was approved by the Local Committee for Ethics in Animal Research in Bydgoszcz, Poland (decision number 41/2021). Additionally, all procedures and methods were performed in accordance with ARRIVE guidelines.

### Animals

As a model species, we used 7-day-old BALB/c mice. Rodents were exposed to moderate postnatal hypoxia at the time which corresponds closely to late prenatal human brain development. Generation of neurons in the newborn rodent brain is largely completed, whereas axonal and dendritic branching is ongoing and synaptogenesis is just beginning. In newborn rodents, the first 20 postnatal days represent the period of rapid differentiation of axons and dendrites^52^. These data indicate that the newborn rodent provides a good model for the developing preterm human brain during the third trimester of gestation^53^.

We used 29 BALB/c mice of both sexes (body mass, 4.8 ± 0.25 g). Dams and litters were housed in individually ventilated cages lined with wood shavings and additional paper bedding for nesting. All animals were kept under controlled temperature (22 ± 1 °C) in a 12:12 h light:dark cycle, starting at 7:00 a.m., with food and water ad libitum and relative humidity 55 ± 10%.

### Induction of hypoxia

We chose the hypoxia-only model because it induces milder injuries and allows to avoid the non-physiological occlusion of the common carotid artery. Additionally, hypoxia-only methods are commonly used to investigate hypoxic brain biochemistry^52,54^ and are established models used to generate seizures in neonatal rats. Our protocol for inducing global hypoxia in neonatal mice was based on the procedure described previously^55,56^.

Individual mice were sealed in 120 mL chambers made of glass, which were submerged in a temperature-controlled water bath. The temperature inside the chambers was regulated at 34.5 ± 0.5 °C to maintain the mouse rectal temperature at 36 ± 0.5 °C. Animals were exposed to a gas mixture of 5% O_2_ and 95% N_2_ (mixed by volume) for 20 min to induce global hypoxia. Gas flow through the chamber was regulated at 400 ml min^–1^ using a needle valve and a mass flow meter (mass flow system, Sable Systems Int., USA). Oxygen concentration in the chamber was monitored using an FMS O_2_ analyzer (Sable Systems Int., USA). Control, normoxic mice were exposed to atmospheric air for the same time and under the same thermal conditions. Throughout the procedure, the rectal temperature of mice was monitored with the T-type thermocouples.

Immediately after hypoxia (H) or control (C) exposure, pups from the same litter were randomly assigned to a vehicle (VEH, 0.9% NaCl) (H/VEH, n = 7; C/VEH, n = 7) or CBD (H/CBD, n = 8; C/CBD, n = 7) treatment. CBD was obtained from a formulation containing 5 mg/ml of CBD (Cayman Chemicals, USA), and was further diluted in saline. After the preparation, it was immediately injected intraperitoneally at a dose of 1 mg kg^–1^ body mass in a volume of 0.1 mL^57^. After the injection, the mice were exposed to atmospheric air at an unchanged temperature for 120 min (recovery period) and then returned to their home cages. 24 h after hypoxia episode animals were decapitated without anesthesia for the measurements of oxidative stress biomarkers. We chose this time point because the secondary energy failure phase, which is related to oxidative stress, occurs 6–48 h after the initial injury^6,7^.

The right hemisphere of the brain was snap-frozen in liquid nitrogen (LN_2_) and stored at − 80 °C until subsequent biochemical analysis. The left hemisphere for morphological analysis and immunohistochemistry was embedded in Tissue-Tek O.C.T. Compound embedding medium (Sakura Finetek, USA), frozen in vapors of LN_2,_ and stored at − 80 °C.

### Markers of oxidative stress

#### Malondialdehyde (MDA) assay

The relative MDA concentration was assessed using a Lipid Peroxidation Assay Kit (Abcam, Great Britain, Catalogue number: ab118970) according to the manufacturer’s instructions. Briefly, 30–50 mg of brain tissue was homogenized in a lysis solution containing butylated hydroxytoluene using an ultrasonic homogenizer (Sonics Vibra-Cell; Sonics & Materials, Inc., USA). The insoluble fraction was removed by centrifugation for 10 min at 13,000×*g*, and the supernatant was used for analysis. For each sample, 200 µl of supernatant was added to 600 µl of thiobarbituric acid (TBA) solution, reconstituted in glacial acetic acid, and then incubated at 95 °C for 60 min in a dry-block thermostat (Bio TDB-100, Biosan, Latvia) and finally cooled immediately on ice for 10 min. The supernatants containing MDA-TBA adducts were added into a 96-well microplate for analysis. Each sample was run in duplicate. Colorimetric changes in the sample were detected using a microplate reader (BioTek Instruments, Inc., Winooski, UT, USA) at 532 nm. The concentration of MDA was expressed in nanomoles per milligram (nmol mg^–1^) of tissue.

#### 8-epi prostaglandin F2α (8-epi PGF2α) assay

Brain 8-epi PGF2α content was determined with an 8-Isoprostane ELISA Kit (Cayman Chemicals, USA, Catalogue number: 516351). The samples were homogenized using an ultrasonic homogenizer (Sonics Vibra-Cell; Sonics & Materials, Inc., USA) in 0.1 M phosphate buffer containing 1 mM ethylenediaminetetraacetic acid and 0.005% butylated hydroxytoluene (1:10 w:v, pH 7.4) and centrifuged. The supernatants were collected and used for analysis. ELISA test was conducted according to standard guidelines provided by the manufacturer. Each sample was run in duplicate. Absorbance was read at 405 nm in a microplate reader. The concentration of 8-epi PGF2α was expressed in picograms per milligram (pg mg^–1^) of tissue.

#### Glutathione (GSH) assay

GSH was determined using a kinetic assay as described by Rahman et al.^58^. The assay is based on the reaction of the sulfhydryl group of glutathione (GSH) with 5,5′-dithiobis-2-nitrobenzoic acid (DTNB) that produces the yellow-colored 5-thio-2-nitrobenzoic acid (TNB) and oxidized glutathione – TNB adduct (G S-TNB). The disulfide product (GS–TNB) is then reduced by glutathione reductase (GR) in the presence of nicotinamide adenine dinucleotide phosphate (NADPH) and GSH is recycled back to the reaction. The rate of TNB production is directly proportional to this recycling reaction, which in turn is directly proportional to the concentration of GSH in the sample. Briefly, the tissue samples were homogenized using an ultrasonic homogenizer (Sonics Vibra-Cell; Sonics & Materials, Inc., USA) in 0.6% sulfosalicylic acid–Triton-X solution, then the homogenate was centrifuged at 8000×*g* for 10 min at 4 °C, and the supernatant was used for analysis. The samples were mixed with DTNB: GR solutions (Sigma‐Aldrich, Germany) and β-NADPH (Sigma‐Aldrich, Germany) and then were measured using a microplate reader at 412 nm (5 readings in total from 0 to 120 s). The reading was repeated twice for each sample. A standard curve made of fresh GSH (Sigma‐Aldrich, Germany) was used to calculate the concentrations of GSH. The concentration of GSH was expressed in micromoles per milligram (µmol mg^–1^) of tissue.

#### Total antioxidant capacity (TAC) assay

The total antioxidant capacity in the brain was determined using a commercial kit (Sigma‐Aldrich, Germany, Catalogue number: MAK187) which measures the concentration of the small molecule and protein antioxidants. According to this method, the reduced Cu^+^ ion chelates with a colorimetric probe, giving a broad absorbance peak at 570 nm, which is proportional to the total antioxidant capacity in Trolox equivalents (a water‐soluble vitamin E analog used as an antioxidant standard). The absorbance at 570 nm was measured using a microplate reader. Each sample was assayed in duplicate. The concentrations of the antioxidants in the samples were then calculated with the value obtained from Trolox standards and results were calculated as nanomoles of Trolox Equivalents per milligram (nmol Trolox Equiv. mg^–1^) of tissue.

### Iron staining

Embedded tissues were frozen sectioned (7 µm thick) and mounted on a poly-L-Lysine coated glass slides. Sections were analyzed for the presence of free iron using a HEMATOGNOST Fe^®^ (Sigma‐Aldrich, Germany, Catalogue number: 112084) staining kit for the detection of free ionic iron in cells according to the manufacturer’s instruction. Briefly, slides were immediately transferred into a working staining solution (equal volumes of 10% potassium ferrocyanide and 10% hydrochloric acid) for 20 min at room temperature. Any ferric ions in the tissue combine with the ferrocyanide and the result is the formation of a bright blue pigment. Subsequently, slides were rinsed in distilled water and then counterstained with nuclear-fast red for 5 min at room temperature. Finally, sections were cleared with xylene and mounted with DPX medium (Sigma‐Aldrich, Germany). The stained sections were examined under a light microscope (Olympus BX41, Olympus Inc., Japan). In the obtained microscopic images, the detected blue spots are iron, and the pink spots are the cellular nuclei.

### Immunofluorescence assay for hypoxia marker (HIF-1α)

Coronal slices (7 μm thick) mounted on a poly-L-Lysine coated glass slides were first immersed in ice-cold acetone for 5 min. Then sections were incubated with the polyclonal rabbit anti-HIF-1α antibody (Novus Biologicals, USA; Catalogue number: NB100-134; 1:50 in1% BSA in PBS) at 4 °C overnight. After rinsing with PBS, the sections were treated with Cy3-conjugated goat anti-rabbit secondary antibody (Thermo Fisher Scientific, USA, Germany; Catalogue number: A10520; 1:200 in PBS) for 1 h at 37 °C. After washing, sections were subjected to DNA staining with Hoechst (Thermo Fisher Scientific, USA, 1:5000 in PBS), rinsed, and mounted in ProLong Gold antifade reagent (Thermo Fisher Scientific, USA). For all incubations, a humidified chamber was used. To ensure comparable results for quantitative measurements, all sections were processed together at the same time under the same conditions. Simultaneous examinations of negative controls (omission of the primary antibody) confirmed the absence of nonspecific immunofluorescent staining. Measurements were done in the hippocampus region. Images were captured with Olympus FluoView™ FV3000 confocal microscope (Olympus Inc., Japan) under consistent conditions of acquisition to ensure comparable results. Five random images from each brain sample were captured within a standard region of interest (ROI), and the level of fluorescence was expressed in arbitrary units (as the product of Area and Mean Gray Value). Before recording the fluorescence intensity, we eliminated the background by adjusting the threshold based on autofluorescence from the negative control. Free iron content and HIF-1α fluorescence level were quantified using Fiji open-source software.

### Statistical analysis

All statistical analyses were done using the SPSS 26.0 package (IBM Corp., Armonk, NY, USA). All data were tested for normality (Shapiro–Wilk test) and homogeneity of variance (Levene test). A Student’s *t*-test for independent samples was used to compare iron deposits in hypoxic animals. All other data were analysed with General Linear Model (GLM), with oxygen conditions and drug treatment as fixed factors, and LSD test with Bonferroni correction for post-hoc comparisons.

### Supplementary Information

Supplementary Information.

## Data Availability

The datasets generated during the current study are available in a Supplementary File.
